# Frank Hastings Hamilton, M. D.

**Published:** 1886-09

**Authors:** 


### FRANK HASTINGS HAMILTON, M. D.

The physical man has passed away, but his good name and fair fame will long live in the affectionate remembrance of his colleagues.

The standard works of Dr. Hamilton are so generally and favorably known to the medical profession as to afford a lasting memorial of his worth. The light shed upon practical surgery by his productions will continue to illumine the pathway of progressive science throughout the future, and give him a prominent place in the constellation of great men whose names shine forth to guide our footsteps. This is not the gleam of a passing meteor or the effulgence of a transient comet, but, like the bright radiance of the sun, the emanations of his intellect will go out into all the world, imparting light to the surgeon for all time.

What Flint left for the advancement of general practice finds a counterpart in the legacy of substantial benefits to surgery by Hamilton. These distinguished men co-operated in building upon a broad basis the grand edifice of science, but time and space will not admit of a proper presentation of the parallel in their respective spheres of duty which all must have observed.

Dr. Hamilton always manifested that straightforward simplicity in his bearing which is the characteristic of great minds, and exhibited that unostentatious presentation of his views which marks the true scientist, while all who came in contact with him in the private relations of life were impressed with his urbanity and courteousness. In the highest and noblest sense of the term, he was a gentleman, and it is hoped that not only his professional attainments, but his personal worth and elevated sentiments may be emulated by all who prize honorable distinction in the medical profession.

### ITEMS.

A WISE dissecting law for Georgia is a necessity.

DR. A. J. WOODWARD, of this city, is on a visit to Cincinnati.

DR. CHAS. I. THOMPSON, of Dixon, Ga., was in Atlanta a few days ago.

DR. A. W. CALHOUN is spending his vacation in the mountains of Northeast Georgia.

PHYSICIANS in Georgia should vote for no man for the legislature who is opposed to a dissecting law.

DR. W. F. WESTMORELAND, JR., after an absence of several weeks in New York, has returned to the city.

DR. HENRY WILE, of this city, has removed his office from 30 Marietta street to his residence, 33 Luckie street.

THE MICHIGAN STATE MEDICAL SOCIETY has donated $500 to the Treasurer of the International Medical Congress.

WE had a pleasant call from Dr. R. H. Taylor, of Griffin, a few days since. The doctor is one of the most progressive men in Georgia.

THE London Hospital Sunday Fund amounts this year to over one hundred and eighty thousand dollars, being $25,000 more than it was last year.

PHYSICIANS in Georgia, do your duty in the coming election by refusing to vote for any candidate for the legislature who is opposed to a dissecting law.

THE little son of Dr. George H. Noble, aged one year, died August 19th at his home in this city. We extend to the bereaved family our sincere sympathy.

DR. F. E. DANIEL, editor *Texas Medical Journal*, has been tendered the Secretaryship of the Texas State Medical Association made vacant by the death of Dr. Burt.

DR. WM. C. DABNEY, of Charlottesville, Va., has been elected to the chair of Practice of Medicine, Obstetrics and Medical Jurisprudence in the Medical Department of the University of Virginia.

DR. W. J. BURT, Secretary of the Texas State Medical Association, died at his home in Austin, July 9th, of acute dysentery. He was one of the most active members of that Association, and his death will be a great loss to that body. Dr. Burt was a Georgian. He formerly lived in Dawson county of this State.

WHILE THE JOURNAL does not propose to go into politics as a business, it will ever oppose the election to the legislature of sentimental cranks and weak-kneed politicians, such as defeated the dissecting bill before the last session of the legislature. The physicians in Georgia are influential, and can do much towards keeping such men at home, and they ought to do it.

THE CENTURY.—This well-known and popular monthly comes to our table regularly, and is always well filled with choice matter. It has few equals and no superiors as a literary magazine.

TWO FEMALE medical students at Paris, the one French and the other American, had a dispute over the relative merits of French and American female physicians. This led to a duel with swords. The American received a slight flesh wound, when both were satisfied.

NEWSPAPER MEDICINE again crops out, this time in the columns of a Boston daily, according to which the stomach of a man who swallowed a partial set of artificial teeth, which lodged in the cardiac orifice, was drawn out through a transverse incision in the left side of his abdomen "and then cut open, when by the insertion of his arm to the elbow, Dr. —— was able to reach and remove the teeth."—*N. Y. Med. Journal.*

The above is in keeping with an account given in a recent number of the New York *World* of the removal of a breast of a lady at one of the hospitals in New York. The exactness of the report shows conclusively that the report was dictated by the doctors, if indeed it was not written by them. Boston is not alone in this newspaper medicine.

SKEPTICISM ABOUT HYDROPHOBIA.—"We have been somewhat surprised," says the *Neurological Review*, "to notice the readiness with which so many condemn the method of M. Pasteur for arresting or preventing hydrophobia. Some have even gone so far as to intimate doubt as to the reality of any such disease. It appears to us, to say the least, curious how it is possible for any one to consider rabid animals, to witness the symptoms they present, the progress of the disease, and finally their death, and doubt whether it actually exists. It is to us almost as much of a surprise to find persons doubting whether there is, or may be, such a disease as hydrophobia in the human species, especially as communicated by the bites of rabid animals. If it is not well established that such a disease as rabies really exists, then, for ourselves, we hardly know what can be considered established. And we have been to a less degree surprised at the readiness with which M. Pasteur and his proposed method of arresting or preventing hydrophobia in man have been condemned, too often by those who have given but little attention either to the character of the man himself or to the course of experimentation upon which his views are based."—*New York Medical Journal.*

THE ECLECTIC.—The September *Eclectic*, published by E. R. Pelton, 25 Bond street, New York, comes again to delight those interested in the best foreign literature. The articles selected for the current issue are valuable, suggestive and entertaining. Leopold Katscher's monograph on Taine is a brilliant study of the French historian and opens the number. There are quite a number of other fine articles of a high standard, and the contents of the number as a whole will be certain to gratify readers of intellectual tastes, a class to which the readers of this magazine distinctly belong.

POISONING BY CHLORATE OF POTASH.—A workman in Vienna, suffering from inflammation of the throat, was given two ounces chlorate of potash with verbal instruction to use it for a gargle; the only instruction upon the prescription was, however, "A coffee spoonful in a glass of water." The patient's wife gave her husband a spoonful at one o'clock and another at two in a medium size tumbler of water and half a spoonful about five and another about six. Abdominal pains and diarrhœa set in shortly after taking the first dose. At 7:30 profuse perspiration came on and about nine sleep. At ten the patient became unconscious, and at one in the morning death took place.—*Medical Press and Circular, August 11th.*

A NOVEL OBSTETRICAL EXPEDIENT.—Dr. Shustoft writes in *Russkaya Meditsina* of April, 1886, that he was called to see a woman who had been in labor five days. The pains had begun well, but had since ceased. Upon examination he saw something black protruding from the anus, and a little pulling brought to light a sausage over seventeen inches long and fourteen inches in circumference. The pains now began again, and the woman was soon delivered of a dead child. Dr. Shustoff found on inquiry that the sausage had been introduced on the recommendation of an old woman of the neighborhood in order to insure the birth of the child by the normal passage. This was probably the old wife's best attempt at supporting the perineum.—*Medical Record.*

DEATH FROM CHLOROFORM.—Lady Flora Wilmot died at Swansea, England, after taking chloroform in a dentist's chair for the extraction of a tooth. The anæsthetic was administered by a physician. The patient had taken chloroform twice before without any bad effects. In all, but two drachms were used. All attempts to restore the patient by the use of nitrite of amyl and artificial respiration were of no avail. The physician remarked immediately after the extraction of the tooth, "I hate giving chloroform for you dentists because you will have your patients sitting up." Both the dentist and the physician were exonerated by the jury which was called to hold an inquest. *Science* comments on this case as follows: "The evidences of the danger in the administration of chloroform are so overwhelming, except in a few cases, that no one is justified, at the present day, in using it in so simple an operation as the extraction of a tooth; and a jury would be doing its full duty in holding responsible for the death of the patient any physician or dentist who administered it in such a case with a fatal result."—*Boston Medical and Surgical Journal.*

ON LESIONS OF THE CORNEA FOLLOWING THE INSTILLATION OF COCAINE.—Numerous instances have now been recorded in which the use of cocaine has been followed by local ill effects. These may be divided into two classes, namely, suppurative panophthalmitis and lesions of the cornea. The former being, in all probability, due to septic infection, cannot be said to depend directly upon the use of cocaine, except in so far as cocaine increases the absorbent properties of the cornea and direct inoculation occurs from impurities in the solution used. The corneal lesions which have been observed are of two kinds: (1) an affection of the epithelial layer only, consisting of vesication or desquamation, and (2) interstitial opacities, often taking the form of a kind of striated keratitis extending from the wound.—*British Medical Journal, August 7.*

A RARE CASE.—A rare departure from the normal uterine development was observed recently, by Dr. J. McF. Gaston, in the person of a colored woman about thirty-five years of age, residing in this city. Within the vulva there was a *cul-de-sac*, which might have received an ordinary hen's egg, without any opening above, and having a density and elacticity corresponding to the vaginal wall. Upon a bimanual examination, neither womb nor ovareis were found either by himself or two colleagues who examined the case with care. The small intestines seemed to rest upon the superior wall, and the descent into the vaginal outlet caused the woman to seek advice and led to this discovery. She supposed that menstruation had been prevented by bathing in cold water when it should have appeared, but there was no subsequent discharge, or indication of a tendency to it at any period since. The mammary development and the external genital organs are normal, and the act of copulation has been attended with the usual orgasm, according to her statement.

A COMPLIMENT TO THE MEDICAL PROFESSION.—In the course of an able article on "Ministerial Quackery" in the *Wesleyan Christian Advocate*, of August 11th, a writer pays the following compliment to the medical profession: It is a noteworthy fact that the medical profession has more perfectly than any other maintained its high and pure standard of excellency, and all the time made steady and substantial progress. There is something significant in this when we remember that this profession has kept its face steadily against all forms of quackery. It recognizes as a great truth that the interests of humanity demand that a physician and his work should be estimated solely by their real worth. The man who advertises his excellencies, or has himself thus advertised, is suspected at once as being shallow, if not fraudulent, and being conscious of his deficiencies and his inability to pass on real merit, he resorts to the newspaper as a means to catch patronage from light heads and the unthinking. The code of ethics of this profession is steadily against all humbuggery, and we note with pleasure that meekness or modesty has not yet been retired from that code. We readily see that a man in this profession is thus kept up on that high plane where his eye is ever fixed upon an ideal that is pure and elevated and worthy of a man.

THE DANGER OF SELF-MEDICATION.—Examples are plentiful of the risk which attends self-medication, even by medical men, who may be supposed cognizant of the potent nature of the remedies employed. The danger is singularly increased when the drug taken is of a narcotic or anæsthetic character, since by its use the faculty of self-preservation is placed in abeyance, and is unable to direct remedial measures when these have become necessary. A very sad case occurred during the past week, when the wife of a physician died in consequence of an overdose of ether, inhaled to relieve asthmatic attacks, to which the deceased lady was liable. The jury very judiciously added a rider, to the effect that they were of opinion that so large a quantity of ether should not have been placed at her command. The lady was away from home at the time, and the reproach, therefore, was addressed to the persons who unadvisedly, if innocently, acceded to her request for a bottle of the drug. It would be some small consolation to think that this and other cases might serve as a lesson to people to use some discretion in employing toxic agents, but unfortunately past experience does not justify any such hope.—*Medical Press and Circular*, *August 11th.*

THE TREATMENT OF MASTITIS BY REST AND COMPRESSION.—We have received a letter addressed by Dr. William H. Doughty, of Augusta, Ga., to Dr. J. A. Post, of Lansing, Mich., and sent by request of the latter gentleman to *The Record* for publication. The writer, referring to Dr. Post's letter published in these columns under date of November 21, 1885, says: "I have just read your defence of the claim of Dr. Ranney, of your city, to priority and originality in the treatment of mastitis by rest and compression, and by the use of the many-tailed bandage. Allow me to throw a little light on the subject of this disputed claim. You trace Dr. Ranney's claim as far back as 1866. This practice has been taught and pursued in this locality for thirty or more years. In 1853 and 1854, when in attendance upon the lectures at the Medical College of Georgia, I was taught by the distinguished Professor of Surgery, the late L. A. Dugas, M. D., LL. D., to adopt and prefer this method of treatment, and, with few exceptions, have employed it ever since. I presume he was the originator of this simple and effective mode of managing this very serious trouble to lying-in women, and do not doubt that a careful examination of the files of the *Southern Medical and Surgical Journal*, then one of the most influential journals of the South, published in this city, and of which he was for many years one of its most distinguished editors, would reveal an editorial or communication on the subject. He not only recommended the many-tailed bandage as the best and simplest, but, almost in the words of Dr. Ranney, advised it 'for the treatment of congested and inflamed breasts, and for the prevention of the secretion of milk when, on account of a still-birth, or death of the child after delivery, or for any other reason, it became necessary to diminish the augmented activity of the circulation, the tendency to tumefaction, inflammation and its results, or to arrest the secretion of milk in the mammæ.' More than this, he specified its negative advantages in retiring the inflamed organ from sight and officious manipulation and filthy applications of every kind by patient or nurse as of no little importance. This mode of treatment is so familiar here as no longer to possess a feature of novelty; indeed, it is rather a matter of surprise that a claim to its discovery should be made in these latter years by any physician from any quarter of the country."—*Medical Record.*

NINTH INTERNATIONAL MEDICAL CONGRESS.—The Ninth International Medical Congress will assemble in the city of Washington, the capital of the United States, on Monday, September 5th, 1887, at 12 o'clock noon, in accordance with the arrangements made at Copenhagen in August, 1884.

The congress will consist of such members of the rugular medical profession as shall have registered and taken out their ticket of admission, and of such other scientific men as the Executive Committee of the congress shall deem it desirable to admit. The books for the registration of members will be open from 9 a. m. to 5 p. m. on Thursday, September 1st, 1887, and on each subsequent day during the session, under the charge of the "*Reception Committee.*" Any member desiring to anticipate this registration can apply by letter to the Secretary General and forward his dues, with his address in full, when a receipt will be returned.

The dues of membership for residents of the United States will be ten dollars ($10.00). There will be no dues for members residing in other countries. Each member will be entitled to receive a copy of the "*Transactions*" of the congress when published by the Executive Committee.

The General Sessions of the Congress will be devoted to the transaction of business and addresses and communications of general scientific interest, by members appointed by the Executive Committee. A printed "programme" of the sessions will be presented to each member on registering. Printed "Order of Business" for each day will also be issued.

The work of the various sections will be directed by the president of the section, and the order will be published in a daily programme for each section. Questions and topics that have been agreed on for discussion in the sections shall be introduced by members previously designated by the titular officers of each section. Members who shall have been appointed to open discussions shall present to the secretaries of the section, in advance, statements of the conclusions which they have formed as a basis for the debate.

Brief abstracts of papers to be read in the sections shall be forwarded to the secretaries of the proper section on or before *April 30th, 1887*. These abstracts shall be treated as confidential communications, and shall not be published before the meeting of the congress. Papers relating to topics not included in the list of subjects proposed by the officers of the sections may be accepted after April 30th, 1887, and any member wishing to introduce a topic not on the regular lists of subjects for discussion shall give notice of the same to the Secretary General at least twenty-one days before the opening of the congress. The titular officers of each section shall decide as to the acceptance of such proposed communications and the time for their presentation. No communication shall be received which has been already published or read before a society.

The official languages of the congress shall be English, French and German. Each paper or address shall be printed in the "*Transactions*" in the language in which it was presented. Preliminary abstracts of papers and addresses shall also be printed in the language in which each is to be delivered. All discussions shall be printed in English.

The officers of the congress and the officers of the sections, including all foreign officers, will be nominated to the congress by the Executive Committee at the opening of the first session. A partial list of the officers to be nominated (except the members of council of the different sections, the list of whom is at present imperfect) is offered herewith.

The Executive Committee cordially invites members of the regular medical profession, and men eminent in the sciences collateral to medicine, in all countries, to participate, in person or by papers, in the work of this great humanitarian assembly. Communications relating to appointments for papers to be read in the congress should be addressed to Dr. John B. Hamilton, Secretary General of the Ninth International Medical Congress, Washington, District of Columbia. All questions or communications connected with the business of the Executive Committee should be addressed to Dr. Henry H. Smith, chairman of the Executive Committee of the Ninth International Medical Congress, Philadelphia, Pennsylvania. Gentlemen named in any position in the congress are requested to notify the chairman of the Executive Committee, as soon as practicable, of any error in the name, title or address in this circular.

Ladies in attendance with members of the congress, and those invited by the "Reception Committee," may attend the General Sessions of the congress when introduced by a member. They will also be invited to attend the social receptions. The Executive Committee reserves the right to invite distinguished persons to any or all the meetings of the congress. The attendance of medical students and others interested in the work of the various sections, or in the general addresses delivered in the congress, will be permitted, on the recommendation of the Secretary General or the officers of a section, on their taking out from the Registration Committee a general ticket of admission, fee one dollar ($1.00); but such persons cannot take part in the proceedings.

All communications and questions relating to the special business of any section must be addressed to the president or one of the secretaries of that section. As many details of the congress and numerous appointments of officers are yet to be completed, other circulars will be issued from time to time as circumstances may demand.—*Extract Circular No. 2.*

THE DOCTOR AS PATIENT.—A little personal experience of the sick bed teaches the doctor many things. He certainly learns that a sick man does not look upon things as a well man does, and his charity towards an invalid's whims is greatly increased. He cannot fail, too, to be touched and softened by the many kind inquiries and pleasant messages that come to him. Busy men come and sit down beside him as though the dearest object of their hearts was to see him recover; men who justly plead bodily infirmity as an excuse against the slightest exertion climb his stairs to express their sympathy, and patients who have seemed thankless and forgetful show that they needed only the opportunity to show their gratitude, and when the sick man resumes his place in life, he is pretty sure to have not merely an increased enjoyment in living, and a better idea of his fellowmen, but also a higher estimate of the value of his own profession.—*Boston Med. and Surg. Journal.*

### METEOROLOGICAL SUMMARY FOR JULY, 1886, STATION, ATLANTA, GA.

| | |
|---|---|
| Mean Temperature, | 76.0 |
| Highest Temperature, July 28th, | 92.0 |
| Lowest Temperature, July 16th, | 58.0 |
| Monthly Range of Temperature, | 34.0 |
| Greatest Daily Range of Temperature, | 25.0 |
| Least Daily Range of Temperature, | 12.0 |
| Mean Daily Range of Temperature, | 18.0 |
| Prevailing Direction of Wind, | West |
| Total movement of wind, | 5,271 miles. |
| Highest Velocity of Wind and Direction, | 19 Miles, N. |
| Total Precipitation. | 2.08 inches. |
| Number of Days on which .01 inch or more of Rain fell | 8.0 |
| Number of Foggy Days | .0 |
| " " Clear " | 14.0 |
| " " Fair " | 17.0 |
| " " Cloudy " | .0 |
| Dates of Thunder-storms, | 2, 3, 5, 6, 7, 8, 9, 10, 11, 14, 21, 31. |

